# The Metabolite Repair Enzyme Phosphoglycolate Phosphatase Regulates Central Carbon Metabolism and Fosmidomycin Sensitivity in Plasmodium falciparum

**DOI:** 10.1128/mBio.02060-19

**Published:** 2019-12-10

**Authors:** Laure Dumont, Mark B. Richardson, Phillip van der Peet, Danushka S. Marapana, Tony Triglia, Matthew W. A. Dixon, Alan F. Cowman, Spencer J. Williams, Leann Tilley, Malcolm J. McConville, Simon A. Cobbold

**Affiliations:** a Department of Biochemistry and Molecular Biology, Bio21 Molecular Science and Biotechnology Institute, University of Melbourne, Parkville, Australia; b School of Chemistry and Bio21 Molecular Science and Biotechnology Institute, University of Melbourne, Melbourne, Australia; c The Walter and Eliza Hall Institute of Medical Research, Parkville, Australia; d Department of Medical Biology, University of Melbourne, Melbourne, Australia; Washington University School of Medicine

**Keywords:** CRISPR, *Plasmodium falciparum*, antimalarial, fosmidomycin, glycolysis, isoprenoid, metabolic regulation, metabolism, metabolomics, parasitology, pentose

## Abstract

Members of the haloacid dehalogenase (HAD) family of metabolite phosphatases play an important role in regulating multiple pathways in Plasmodium falciparum central carbon metabolism. We show that the P. falciparum HAD protein, phosphoglycolate phosphatase (PGP), regulates glycolysis and pentose pathway flux in asexual blood stages via detoxifying the damaged metabolite 4-phosphoerythronate (4-PE). Disruption of the P. falciparum *pgp* gene caused accumulation of two previously uncharacterized metabolites, 2-phospholactate and 4-PE. 4-PE is a putative side product of the glycolytic enzyme, glyceraldehyde-3-phosphate dehydrogenase, and its accumulation inhibits the pentose phosphate pathway enzyme, 6-phosphogluconate dehydrogenase (6-PGD). Inhibition of 6-PGD by 4-PE leads to an unexpected feedback response that includes increased flux into the pentose phosphate pathway as a result of partial inhibition of upper glycolysis, with concomitant increased sensitivity to antimalarials that target pathways downstream of glycolysis. These results highlight the role of metabolite detoxification in regulating central carbon metabolism and drug sensitivity of the malaria parasite.

## Abstract (precis)

The malaria parasite has a voracious appetite, requiring large amounts of glucose and nutrients for its rapid growth and proliferation inside human red blood cells. The host cell is resource rich, but this is a double-edged sword; nutrient excess can lead to undesirable metabolic reactions and harmful by-products. Here, we demonstrate that the parasite possesses a metabolite repair enzyme (PGP) that suppresses harmful metabolic by-products (via substrate dephosphorylation) and allows the parasite to maintain central carbon metabolism. Loss of PGP leads to the accumulation of two damaged metabolites and causes a domino effect of metabolic dysregulation. Accumulation of one damaged metabolite inhibits an essential enzyme in the pentose phosphate pathway, leading to substrate accumulation and secondary inhibition of glycolysis. This work highlights how the parasite coordinates metabolic flux by eliminating harmful metabolic by-products to ensure rapid proliferation in its resource-rich niche.

## INTRODUCTION

Plasmodium falciparum is the major cause of malaria, a disease that causes ∼445,000 deaths each year and disproportionally affects people living in developing countries (1). The symptoms of malaria arise from the progressive ∼48-h cycle of parasite invasion of host red blood cells (RBCs), rapid growth and asexual replication, and subsequent host cell rupture. RBCs provide the resource-rich environment required to support the aggressive proliferation of the intracellular parasite. Glucose is the principal carbon source for P. falciparum asexual blood stages, which is metabolized by glycolysis for generation of ATP and biosynthetic precursors. However, high rates of glycolysis can generate toxic metabolic end products, such as methylglyoxal, which can lead to increased oxidative stress, pernicious chemical modification, and denaturation/inactivation of proteins, lipids, and DNA (2–4). Therefore, it is likely that P. falciparum has evolved mechanisms to regulate glycolysis under nutrient-excess conditions to help curtail these toxic effects.

P. falciparum expresses a limited number of transcription factors and has minimal nutrient-stimulated transcriptional control (5–8), indicating that regulation of central carbon metabolism occurs primarily at the posttranscriptional level. Members of the haloacid dehalogenase (HAD) family of metabolite phosphatases have been shown to have a role in regulating glycolysis in P. falciparum asexual stages. The first member of this family to be functionally characterized was HAD1, identified in a screen for P. falciparum mutant lines that were resistant to the isoprenoid biosynthesis inhibitor fosmidomycin (9). HAD1 exhibited broad *in vitro* phosphatase activity against a range of sugar phosphates and triose phosphates that are connected to glycolysis, suggesting that it has a role in the promiscuous dephosphorylation of glycolytic intermediates and restricts glycolytic flux via substrate depletion. Mutational inactivation of HAD1 leads to increased glycolytic flux and the flow of intermediates into anabolic pathways, such as isoprenoid biosynthesis, with an associated increase in fosmidomycin resistance. Interestingly, a second HAD enzyme, HAD2, was identified in the same screen and exhibited *in vitro* phosphatase activity against glycolytic intermediates, suggesting that HAD enzymes regulate multiple pathways in P. falciparum central carbon metabolism (10).

A third member of the P. falciparum HAD family shares homology to the enzyme phosphoglycolate phosphatase (PGP), which is involved in regulating intracellular levels of several metabolites, including glycerol-3-phosphate, 2-phosphoglycolate, 2-phospholactate, and 4-phosphoerythronate (4-PE) (11, 12). 2-Phospho-lactate and 4-PE are minor side products of the high-flux reactions catalyzed by pyruvate kinase and glyceraldehyde-3-phosphate dehydrogenase (GAPDH), respectively (11). Thus, PGP may act as a metabolite repair enzyme that removes metabolites that would otherwise accumulate and inhibit key enzymes in central carbon metabolism (13, 14). Previous work has suggested that PGP in P. falciparum dephosphorylates a range of metabolites, including thiamine pyrophosphate, and its homolog in P. berghei was reported to be essential for regulating 2-phosphoglycolate and 2-phospholactate levels (15, 16). Here, we establish in P. falciparum the role of PGP in metabolite repair and maintaining central carbon metabolic activity. Disruption of PGP leads to the accumulation of 2-phospholactate and 4-PE in the parasite, which in turn leads to the 4-PE-mediated inhibition of the essential pentose phosphate pathway (PPP) enzyme, 6-phosphogluconate dehydrogenase (6-PGD). Unexpectedly, this inhibition initiates a feedback effect whereby the substrate of 6-PGD accumulates and inhibits the glycolytic enzyme, glucose-6-phosphate isomerase, leading to glycolytic blockage and redirection of carbon into the PPP. These data highlight the importance of metabolic repair enzymes for maintaining metabolic homeostasis in these parasites in the absence of canonical transcriptional regulatory pathways.

## RESULTS

### P. falciparum PGP is a cytoplasmic protein required for normal growth during asexual intraerythrocytic development.

**FIG 1:**
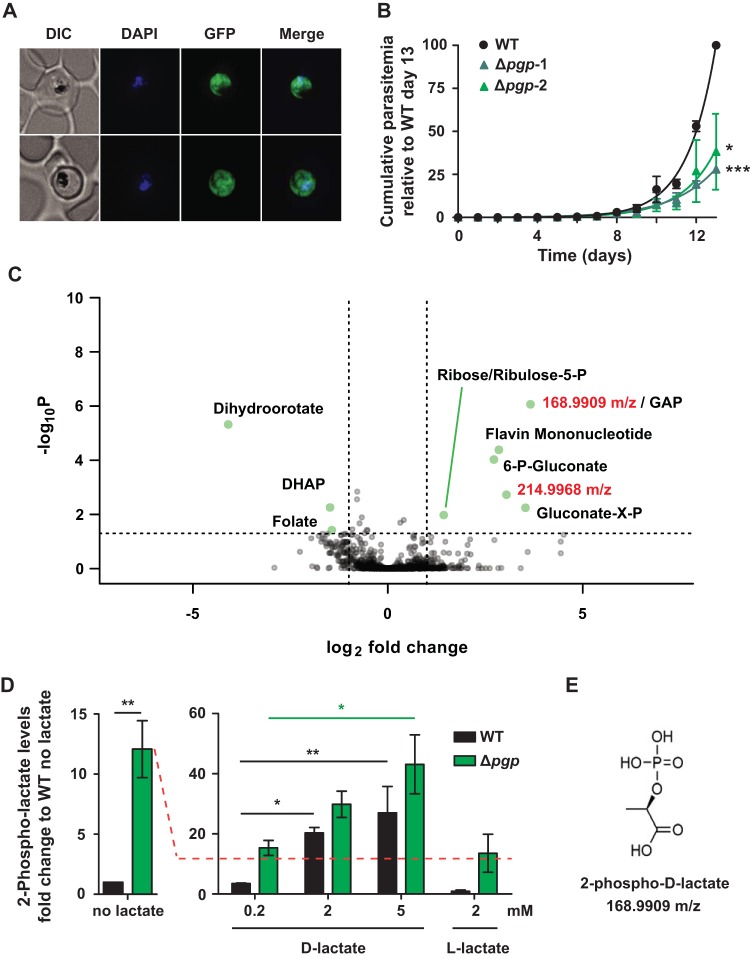
PGP is required for normal growth of P. falciparum asexual stages, and Δ*pgp* mutant parasites selectively accumulate metabolites in the PPP and two nonstandard metabolites. (A) Fluorescence microscopy of live PGP-GFP-infected RBCs. Infected RBCs were labeled with DAPI and visualized by differential interference contrast (DIC) and fluorescence microscopy. GFP fluorescence was present throughout the cytoplasm. (B) The asexual growth of Δ*pgp* strain- versus wild-type (WT)-infected RBCs was monitored daily over a 13-day period by flow cytometry following SYTO61 labeling. Data are presented as the mean ± standard errors of the mean (SEM) cumulative parasitemia normalized to the WT day 13 data point (100%) from three independent experiments. Statistical significance was determined using a paired Student's *t* test at day 13 (*, *P* < 0.05; ***, *P* < 0.001). (C) Intracellular metabolite profiling of WT- and Δ*pgp* mutant-infected RBCs by LC-MS. Individual metabolites are plotted as fold change (log_2_) versus –log_10_(*P*) for the Δ*pgp* mutant parasites compared to WT parasites. Annotated metabolites were verified using standards, with the exception of gluconate-X-P (where the position of the P is unknown). Ribose-5-P and ribulose-5-P coelute under the chromatography conditions used. Two peaks of unknown identity, *m/z* 168.9909 and *m/z* 214.9968, also increased in the mutant line. (D) WT- and Δ*pgp* mutant-infected RBCs were incubated with different concentrations of l- or d-lactate, and intracellular levels of 2-phospholactate were measured by GC-MS. Data are presented as fold change versus the WT (no lactate) condition. Data are presented as the means ± SEM from three independent experiments performed on different days. Statistical significance was determined using an unpaired Student's *t* test for the condition of WT with no lactate versus Δ*pgp* mutant (**, *P* < 0.01), and one-way analysis of variance (ANOVA) was used for all lactate stimulation conditions (*, *P* < 0.05; **, *P* < 0.01; all other comparisons were nonsignificant). (E) Structure of 2-phospho-d-lactate (C_3_H_7_O_6_P).

The P. falciparum gene PF3D7_0715000 (here referred to as PGP) shares homology with the phosphoglycolate phosphatase (PGP) family of HAD enzymes, which are involved in regulating the intracellular levels of several phosphometabolites generated as side products of glycolysis. In particular, the P. falciparum PGP is predicted to have the four characteristic HAD motifs found in PGPs from other eukaryotes (see Fig. S1 in the supplemental material) (17). Consistent with previous studies (16), a PGP-green fluorescent protein (GFP) fusion protein is exclusively located in the cytoplasm of asexual blood stages (Fig. 1A) and is readily extracted in either phosphate-buffered saline-sodium dodecyl sulfate (PBS-SDS) or radioimmunoprecipitation assay (RIPA) buffer-SDS (Fig. S2A).

10.1128/mBio.02060-19.1 FIG S1 PGP shares homology with other described PGP enzymes. Clustal W alignment (42) of P. falciparum PGP (PF3D7_0715000) with known sequences of yeast Pho13 (YDL236W), mouse AUM (Q8CHP8), and human PGP (NC_000016). The four characteristic HAD motifs (17), framed in red (I to IV), appear to be highly conserved between the compared species. Download FIG S1, PDF file, 0.3 MB. Copyright © 2019 Dumont et al. 2019 Dumont et al. This content is distributed under the terms of the Creative Commons Attribution 4.0 International license.

10.1128/mBio.02060-19.2 FIG S2 PGP is a cytosolic protein and molecular characterization of ΔPGP parasites. (A) Western blot of PGP-GFP (PGP)- and WT-infected RBC lysates probed with anti-GFP and anti-GAPDH (loading control). Samples were extracted in either PBS or RIPA buffer. This blot is representative of three independent experiments. (B) Schematic of the ΔPGP cloning strategy. CDS, coding sequence; UTR, untranslated regions; hDHFR, resistance cassette; (k)bp, (kilo)base pairs. Striped boxes, homology arms; short black line, guide RNA; dotted line between arrows, fragment used for genetic confirmation of the knockout. (C) Genetic confirmation of the disruption of the *pgp* gene by PCR on genomic DNA. A 1-kb DNA ladder was used as a reference. Download FIG S2, PDF file, 0.9 MB. Copyright © 2019 Dumont et al. 2019 Dumont et al. This content is distributed under the terms of the Creative Commons Attribution 4.0 International license.

*In vivo* characterization of PGP was performed via CRISPR/Cas9 disruption of the *pgp* gene (Fig. S2B) (18). Following transfection, the parasite population was immediately split in two and maintained as independent populations. PGP-disrupted P. falciparum mutants (referred to as Δ*pgp*-1 and Δ*pgp*-2 mutants) were recovered, and loss of the gene was confirmed by PCR (Fig. S2C). Both parasite lines presented a significant decrease in growth rate under standard growth conditions (Fig. 1B). The NF54 parental line exhibited a doubling time of 1.04 ± 0.12 days, whereas Δ*pgp*-1 and Δ*pgp*-2 lines had doubling times of 1.48 ± 0.24 days (*P = *0.0005) and 1.21 ± 0.03 days (*P = *0.039), respectively. This growth defect was evident in a second and independently generated 3D7-*Δpgp* line (Fig. S3) and was independent of the potential role of PGP in vitamin B_1_ biosynthesis (16), as the medium was supplemented with vitamin B_1_, among other vitamins. A comprehensive metabolomic analysis of parental (NF54) and *Δpgp* mutant parasite lines was subsequently undertaken to determine if loss of PGP was associated with changes in central carbon metabolism. Trophozoite-infected RBCs were magnetically enriched (>95% parasitemia), and extracted metabolites were analyzed by liquid chromatography-mass spectrometry (LC-MS). The mutant parasite line showed selective increases in metabolites associated with the pentose phosphate pathway (6-phosphogluconate, ribose/ribulose-5-phosphate), flavin mononucleotide, and two unknown peaks with mass-to-charge ratios (*m/z*) of 168.9909 and 214.9968 (Fig. 1C). The unknown peak at *m/z* 168.9909 had the same *m/z* as the glycolytic intermediates, glyceraldehyde phosphate (GAP) and dihydroxyacetone phosphate (DHAP), but different chromatographic retention times from authentic GAP and DHAP standards on LC-MS or gas chromatography-mass spectrometry (GC-MS). This peak also has the same predicted *m/z* as phospholactate. To assess this possibility, a racemic mixture of 2-phospho-dl-lactate was synthesized and analyzed by GC-MS. The P. falciparum *m/z* 168.99 peak had the same GC-MS retention time and mass spectrum as the authentic 2-phospho-dl-lactate (Fig. S4). To distinguish between the two possible enantiomers, parasite-infected RBCs were incubated in the presence of either l- or d-lactate and the intracellular level of 2-phospholactate determined by targeted GC-MS analysis. Consistent with the untargeted analysis, the intracellular level of 2-phospholactate increased 12-fold (±2.4) in the Δ*pgp* line compared to the parental wild-type (WT) line (Fig. 1D, left). Addition of 2 mM l-lactate (the major enantiomer generated by glycolysis) to WT or Δ*pgp* parasite cultures had no effect on the intracellular levels of 2-phospholactate (Fig. 1D, right). In contrast, incubation of either WT or Δ*pgp* parasites with d-lactate resulted in a marked increase in 2-phospholactate. Taken together, these data show that PGP is involved in dephosphorylating 2-phospholactate, and this nonstandard metabolite can be generated via d-lactate, resulting in 2-phospho-d-lactate (Fig. 1E), in addition to the more conventional l-lactate source.

10.1128/mBio.02060-19.3 FIG S3 P. falciparum Δ*pgp* clones exhibit a growth defect in culture. A second P. falciparum Δ*pgp* line (in 3D7 background) was independently generated, and the growth rate was assessed at day 13 by microscopy. Data are presented as the percent parasitemia from individual biological replicates. Download FIG S3, PDF file, 0.8 MB. Copyright © 2019 Dumont et al. 2019 Dumont et al. This content is distributed under the terms of the Creative Commons Attribution 4.0 International license.

10.1128/mBio.02060-19.4 FIG S4 Identification of 2-phospho-d-lactate in wild-type parasites. (A) GC-MS spectrum of 2-phospholactate with fingerprint signature. (B) 3D7 WT-infected RBCs were incubated with d-lactate (1 mM) or left untreated (iRBC condition), and increasing concentrations of a pure 2-phospholactate standard were spiked into cell extracts. The *y* axis represents the arbitrary ion counts, and the *x* axis represents the retention time, in minutes, for the 2-phospholactate peak. (C) GC-MS spectrum of 2-phospholactate present in P. falciparum-infected RBCs. Download FIG S4, PDF file, 0.9 MB. Copyright © 2019 Dumont et al. 2019 Dumont et al. This content is distributed under the terms of the Creative Commons Attribution 4.0 International license.

The P. falciparum *m/z* 214.9968 peak had the expected *m/z* of 4-phosphoerythronate (4-PE; expected *m/z* 214.9962). 4-PE is not an intermediate in canonical metabolic pathways but can be generated by GAPDH acting on the pentose phosphate pathway intermediate erythrose-4-phosphate instead of its preferred substrate, glyceraldehyde-3-P (GAP) (11). The P. falciparum *m/z* 214.9968 peak was subsequently shown to have the same MS spectrum and GC-MS retention time as authentic 4-PE, confirming its identity (see Fig. 3A and Table S1). Together, these data indicate that P. falciparum PGP has a role in regulating the intracellular levels of several noncanonical phosphometabolites generated by high-flux reactions in glycolysis.

10.1128/mBio.02060-19.8 TABLE S1 Repertoire of the metabolites of interest and analytical GC-MS features. Highlight of the quantified ion and retention time used for identifying specific metabolites in all GC-MS-based assays presented in the manuscript. Download Table S1, PDF file, 0.04 MB. Copyright © 2019 Dumont et al. 2019 Dumont et al. This content is distributed under the terms of the Creative Commons Attribution 4.0 International license.

### Phospholactate is a product of the methylglyoxal pathway.

**FIG 2:**
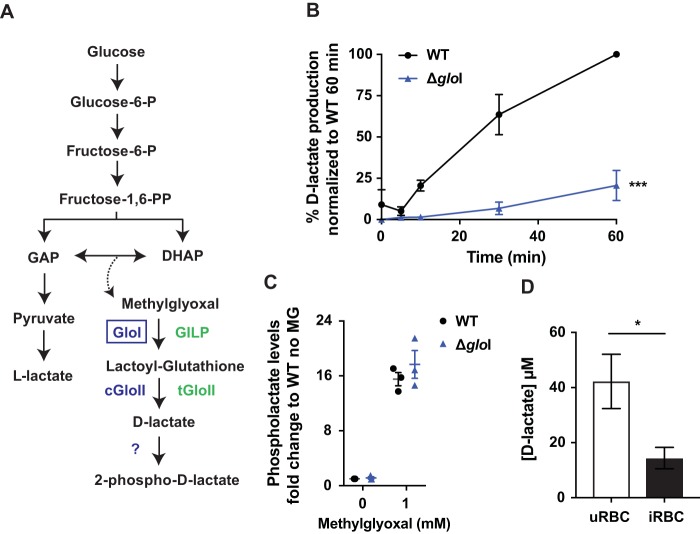
2-Phospho-d-lactate is generated via the glyoxalase pathway. (A) Schematic of the methylglyoxal detoxification system in P. falciparum. GloI and cGloII catalyze the conversion of methylglyoxal to d-lactate. P. falciparum also expresses a GloI-like protein (GILP) and tGloII, which are both targeted to the apicoplast. (B) *In vitro* production of d-lactate in Δ*gloI* parasites versus WT parasites. Data are presented as the percentage of d-lactate production normalized to the WT 60-min time point (100%) after experimental background subtraction. Data are presented as the means ± SEM from three independent experiments performed on different days, and statistical significance was determined using an unpaired Student's *t* test at the 60-min time point (***, *P* < 0.001). (C) WT- and Δ*gloI*-infected RBCs were incubated with 0 or 1 mM methylglyoxal (MG), and the intracellular abundance of 2-phospholactate was measured by GC-MS. Data are presented as fold change relative to the condition of WT with 0 mM methylglyoxal. Data are presented as the means ± SEM from three independent experiments performed on different days. (D) Extracellular d-lactate excretion was measured in uninfected RBCs (uRBC) and WT-infected RBCs (iRBC) after sample deproteination using a d-lactate plate assay (Cayman Chemicals). Cultures were set at 2.5% hematocrit (and 1.8% to 4% parasitemia for infected RBC). Medium was collected after 26 to 29 h of culture (ring to trophozoite stages for infected RBC). Data are presented as the means ± SEM from four independent repeats collected on different days, and statistical significance was determined using an unpaired Student's *t* test (*, *P < *0.05).

In animal cells, 2-phospho-l-lactate is thought to be a by-product of the terminal glycolytic enzyme pyruvate kinase (11). The finding that P. falciparum phospholactate is derived from the d-stereoisomer of lactate indicates that an additional pathway for synthesizing this intermediate must operate. The only pathway known to generate d-lactate in P. falciparum is the methylglyoxal pathway, which is required for detoxification of methylglyoxal formed by nonenzymatic elimination of phosphate from the glycolytic triose phosphates GAP and DHAP (3, 19). Methylglyoxal is converted to *S*-d-lactoyl-glutathione by glyoxalase I (GloI) and then metabolized to d-lactate by glyoxalase II (GloII) (2) (Fig. 2A). To investigate whether the d-lactate generated in this pathway is converted to 2-phospho-d-lactate, we disrupted the cytoplasmic *gloI* gene (PF3D7_1113700) using the CRISPR/Cas9 system (Fig. S5A) (18). Disruption of *gloI* was confirmed by PCR (Fig. S5B). Analysis of lysates of saponin-purified P. falciparum trophozoites indicated that the Δ*gloI* line had greatly reduced GloI activity *in vitro*, as measured by conversion of methylglyoxal and glutathione to d-lactate by GC-MS (Fig. 2B and Table S1). Specifically, the Δ*gloI* parasites exhibited an 80% reduction in d-lactate production after 60 min (WT, 100%; Δ*gloI *parasites, 20.6% ± 9.1%).

10.1128/mBio.02060-19.5 FIG S5 Molecular characterization of Δ*gloI* parasites. (A) Schematic of the Δ*gloI* cloning strategy. CDS, coding sequence; UTR, untranslated regions; hDHFR, resistance cassette; (k)bp, (kilo)base pairs. Striped boxes, homology arms; short black line, guide RNA; dotted line between arrows, fragment used for genetic confirmation of the knockout. (B) Genetic confirmation of the disruption of the *gloI* gene by PCR on genomic DNA. The single band at 1.1 kb in the knockout line only matches the expected 1,145-bp PCR fragment. A 1-kb DNA ladder was used as a reference. Download FIG S5, PDF file, 0.4 MB. Copyright © 2019 Dumont et al. 2019 Dumont et al. This content is distributed under the terms of the Creative Commons Attribution 4.0 International license.

To investigate whether 2-phospholactate is synthesized by the methylglyoxal pathway, parental WT and Δ*gloI* parasite cultures were suspended in medium containing methylglyoxal (1 mM, 1 h, and 37°C), and levels of 2-phospholactate were measured by GC-MS. Addition of methylglyoxal led to a 15-fold increase in 2-phospholactate levels, indicating that this metabolite is the end product of the methylglyoxal pathway (Fig. 2C). Strikingly, similar levels of 2-phospholactate were present in both WT and Δ*gloI* parasites, before and after addition of methylglyoxal, indicating that 2-phospholactate also could be derived from l-lactate via nonpreferred pyruvate kinase activity. Alternatively, methylglyoxal may be converted to d-lactate by the RBC methylglyoxal pathway and d-lactate subsequently imported by the parasite and converted to 2-phospho-d-lactate. Regardless, these studies suggest that 2-phospholactate is generated by the direct phosphorylation of d-lactate produced by either the host cell or the parasite methylglyoxal detoxification pathways.

We hypothesized that d-lactate is converted to 2-phospho-d-lactate to prevent its secretion alongside l-lactate. Consistent with this proposal, analysis of the culture medium of uninfected and infected RBCs showed that secretion of d-lactate was reduced by ∼75% in the latter, indicating that d-lactate is sequestered within the parasite as 2-phospho-d-lactate (Fig. 2D). A previous study indicated that d-lactate secretion increased following erythrocyte infection (19). However, we found that deproteinization of samples was required to remove residual l-lactate dehydrogenase activity in the culture supernatant samples. Otherwise, the activity would have interfered with the coupled d-lactate dehydrogenase-NADH-based fluorescence assay. We speculate that without this step, fluorescence readings were artificially high in this previous study.

### Loss of PGP leads to increased flux through the pentose phosphate pathway.

**FIG 3:**
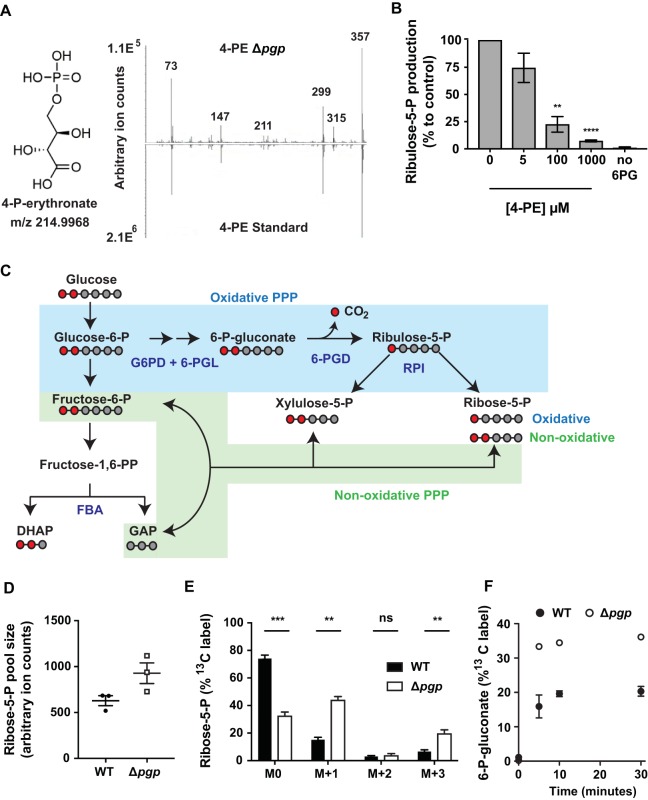
Loss of PGP leads to inhibition of 6-PGD and enhanced flux through the oxidative PPP. (A) Chemical structure of 4-PE (C_4_H_9_O_8_P) and GC-MS confirmation of 4-PE in P. falciparum. (B) Synthetic 4-PE was added to lysates of saponin-purified trophozoites, together with 6-phosphogluconate (6-PG), and the activity of 6-PGD was assessed by measurement of production of ribulose-5-P by GC-MS. Data are presented as percentages of the no 4-PE condition, with means ± SEM from four to five independent experiments performed on different days, and statistical significance was determined using one-way ANOVA in comparison to the condition of 0 mM 4-PE (**, *P < *0.01; ****, *P < *0.0001). (C) Schematic of the pentose phosphate pathway (PPP) in P. falciparum. Where relevant, carbon backbones are presented as gray (unlabeled) or red (^13^C-labeled) circles. The relative activity of the oxidative and nonoxidative PPP was monitored with 1,2-^13^C_2_-glucose incorporation into ribose-5-P, with one and two labeled carbons corresponding to oxidative and nonoxidative PPP activity, respectively. (D and E) The ribose-5-P pool sizes (D) and percent label into ribose-5-P following 1,2-^13^C_2_-glucose labeling (E). M + 1 represents the fraction of ribose-5-P derived from the oxidative arm, M + 2 represents the contribution of the nonoxidative arm, and M + 3 comprises both oxidative and nonoxidative labeling. Data are presented as the means ± SEM from three independent experiments performed on different days. Statistical significance was determined using unpaired *t* testing (*ns*, nonsignificant; **, *P < *0.01; ***, *P* < 0.001). (F) The percent label into 6-phosphogluconate (6-P-gluconate) following 30 min of ^13^C_6_-glucose incorporation (1:1 ^12^C:^13^C). Data are presented as the means ± SEM from three independent experiments performed on different days.

Loss of P. falciparum PGP was associated with a marked increase in 4-PE (Fig. 1C), which has been shown to inhibit enzymes in the pentose phosphate pathway (PPP), including 6-phosphogluconate dehydrogenase (6-PGD) (11, 20). We observed an increase in intracellular levels of 6-phosphogluconate, the substrate of 6-PGD, in Δ*pgp* parasites, consistent with 4-PE accumulation inhibiting 6-PGD in the parasite (Fig. 1C). To determine if 4-PE inhibits 6-PGD directly, we measured the *in vitro* activity of 6-PGD in lysates of saponin-lysed trophozoite-infected RBCs in the absence or presence of 4-PE. Lysates were incubated with 6-phosphogluconate and increasing concentrations of 4-PE, and the conversion of 6-phosphogluconate to ribulose-5-P was measured by GC-MS (Table S1). Significant inhibition was observed at 100 μM (>75% inhibition; *P < *0.01), and complete inhibition occurred with 1 mM 4-PE (Fig. 3B). Neither erythronate nor 2-phospholactate exhibited any inhibitory effects (Fig. S6). These results suggest that the accumulation of 4-PE in micromolar amounts in Δ*pgp* parasites inhibits the activity of 6-PGD. The intracellular concentrations of 4-PE can reach 20 to 30 μM in HCT116 cells when *pgp* is disrupted (11), suggesting that the level of inhibition observed in these assays is physiologically relevant.

10.1128/mBio.02060-19.6 FIG S6 Inhibition of 6-phosphogluconate dehydrogenase is specific to 4-phosphoerythronate. 6-Phosphogluconate dehydrogenase *in vitro* activity was tested by incubating saponin-isolated trophozoite parasites with 6-phosphogluconate and measurement of the ribulose-5-P product by GC-MS. Parasite lysates were incubated with (left to right) no 4-PE, 1 mM 2-phospholactate (+Plac), 1 mM erythronate (+Ery), no NADP, and no reaction buffer (lysate). Results are normalized to the no 4-PE condition (100%). Data are presented as the means ± SEM from three independent experiments performed on different days. Download FIG S6, PDF file, 0.1 MB. Copyright © 2019 Dumont et al. 2019 Dumont et al. This content is distributed under the terms of the Creative Commons Attribution 4.0 International license.

Paradoxically, metabolite profiling indicated that levels of intermediates downstream of 6-PGD were also elevated in Δ*pgp* parasites (Fig. 1C, ribose-5-P/ribulose-5-P). An increase in the pool size of these pentose-phosphates may reflect increased flux through the nonoxidative PPP and/or a compensating increase in flux through the oxidative PPP (overcoming 4-PE inhibition of 6-PGD). To address this question directly, enriched WT- and Δ*pgp* mutant-infected RBCs were metabolically labeled with 1,2-^13^C_2_-glucose to measure flux through both arms of the PPP. Catabolism of 1,2-^13^C_2_-glucose through the oxidative arm of the PPP leads to loss of ^13^C on carbon-1 of 6-phosphogluconate (as carbon dioxide), as this intermediate is converted to 1-^13^C-ribose-5-P (Fig. 3C, M + 1). In contrast, conversion of 1,2-^13^C_2_-glucose to ribose-5-P via the nonoxidative pathway does not involve a decarboxylation step, resulting in 1,2-^13^C_2_-ribose-5-P (Fig. 3C, M + 2).

Purified WT- and Δ*pgp* mutant-infected RBCs were incubated with 1,2-^13^C_2_-glucose for 30 min at 37°C, and ^13^C enrichment in ribose-5-P was determined by GC-MS. Ribose-5-P levels were elevated in Δ*pgp* parasites (Fig. 3D and Table S1), consistent with the results of the initial metabolomic analyses (Fig. 1C). Ribose-5-P turnover was significantly increased in the Δ*pgp* mutant (Fig. 3E, as indicated by the greater M0 depletion in Δ*pgp* parasites) due to increased M + 1 ^13^C-ribose-5-P production (and M + 3 ^13^C-ribose-5-P, likely derived from mixing of carbon derived from the oxidative and nonoxidative arms through transketolase), consistent with increased oxidative PPP flux. This counterintuitive result suggests that partial inhibition of 6-PGD in the Δ*pgp* parasite lines, due to the accumulation of 4-PE, causes increased flux through 6-PGD and the oxidative pentose phosphate pathway. ^13^C_6_-glucose incorporation into 6-phosphogluconate was elevated in Δ*pgp* parasite cultures compared to that of the WT (Fig. 3F, 33.3% ± 0.4% and 15.9% ± 3.3% labeled at 5 min), consistent with increased flux into the oxidative pentose phosphate pathway.

**FIG 4:**
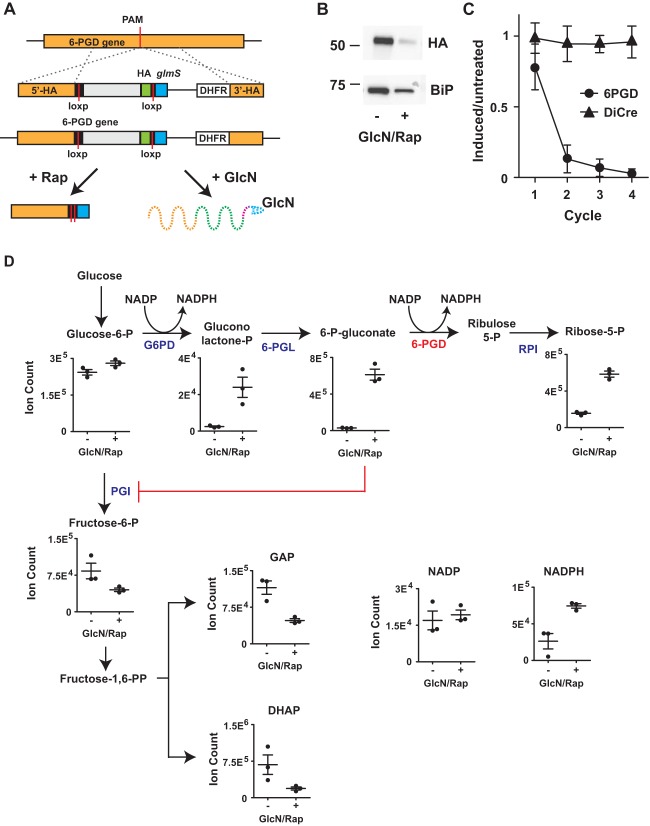
6-PGD disruption leads to glycolytic inhibition and increased oxidative PPP flux. (A) Inducible disruption of 6-PGD via DiCre-dependent gene excision and *glmS* ribozyme-mediated transcript decay. (B) Confirmation of 6-PGD depletion by Western blotting following rapamycin (Rap; 100 nM) and glucosamine (GlcN; 2.5 mM) addition. HA-tagged 6-PGD was detected at the expected size (anti-HA), and anti-P. falciparum BiP was used as a loading control. Indicated sizes are in kilodaltons. (C) Cell viability of DiCre-6-PGD and the parental DiCre lines in the presence or absence of rapamycin (100 nM) and glucosamine (2.5 mM), over four replication cycles. Flow cytometry was performed using SYTO61 labeling, and the data are presented as the treated/untreated ratio, with means ± SEM from three independent experiments. (D) Metabolic phenotype of 6-PGD depletion in trophozoite-stage infected RBCs. Metabolite pools are presented as mean arbitrary ion counts (±SEM) from three independent experiments.

The paradoxical increase in oxidative pentose phosphate pathway flux following partial inhibition of 6-PGD and accumulation of 6-phosphogluconate could reflect feedback stimulation due to a blockage of glycolysis. To specifically investigate the role of 6-PGD in regulating glycolytic flux, we targeted the gene encoding 6-PGD for inducible disruption. Note that 6-PGD is predicted to be essential (21). We genetically modified a stably expressing DiCre recombinase 3D7 line using CRISPR/Cas9 and replaced the 3′ end of the 6-PGD gene with a recodonized version flanked by two LoxP sites and the ribozyme *glmS* (Fig. 4A), as described by M.-L. Wilde et al. (43). This modified gene undergoes DiCre-mediated excision when rapamycin is added and will also undergo ribozyme-mediated transcript degradation when glucosamine is present. Integration of the transfected construct was verified via PCR (Fig. S7). A hemagglutinin (HA) tag also was introduced to monitor protein reduction, and an HA-specific band was detected at the expected size of 6-PGD (56 kDa when combined with the HA tag) (Fig. 4B). The HA-specific band was depleted following addition of 100 nM rapamycin and 2.5 mM glucosamine. Inducible 6-PGD disruption also led to a rapid drop in parasite viability compared to that of the parental 3D7-DiCre line in the presence of 100 nM rapamycin and 2.5 mM glucosamine (Fig. 4C), suggesting that 6-PGD is essential for parasite asexual development.

10.1128/mBio.02060-19.7 FIG S7 PCR confirmation of integration of a recodonized 6-PGD. PCR of gDNA extracted from 6-PGD transfectants and the parental DiCre line. Int, PCR products obtained using oligonucleotides SC108 and SC110, specific for integration of the rescue template described in Table S3. NI, PCRs using SC108 and SC109, which is specific for nonintegration of the native *6-pgd* locus. Download FIG S7, PDF file, 0.8 MB. Copyright © 2019 Dumont et al. 2019 Dumont et al. This content is distributed under the terms of the Creative Commons Attribution 4.0 International license.

To analyze the metabolic phenotype of reduced 6-PGD expression, parasites were exposed to rapamycin and glucosamine for one cycle to induce 6-PGD depletion while avoiding any cell death-associated metabolic phenotype. Trophozoite-stage DiCre-6-PGD parasites (in the presence or absence of rapamycin and glucosamine) were magnetically enriched and metabolites extracted for LC-MS analysis. 6-PGD-disrupted parasites accumulated pentose phosphate intermediates upstream of 6-PGD (6-phosphogluconolactone and 6-phosphogluconate) but also downstream of 6-PGD (ribose/ribulose-5-P) (Fig. 4D), similar to the phenotype observed in the Δ*pgp* line (Fig. 1C and 3D). As the oxidative PPP is the primary source of NADPH regeneration in P. falciparum, increased flux into this pathway would be expected to be associated with increased NADPH levels. Indeed, NADPH levels were significantly elevated when 6-PGD expression was knocked down, consistent with feedback stimulation of this pathway following accumulation of 6-phosphogluconate (Fig. 4D). Moreover, it was clear that glycolytic intermediates below glucose-6-phosphate isomerase were substantially reduced in 6-PGD-disrupted parasites. Collectively, these results show that accumulation of 6-phosphogluconate, due to partial inhibition of 6-PGD (through accumulation of 4-PE in the Δ*pgp* parasites or direct knockdown), leads to inhibition of upper glycolysis and increased flux into the oxidative PPP.

### Loss of PGP reduces glycolytic flux and increases sensitivity to fosmidomycin.

**FIG 5:**
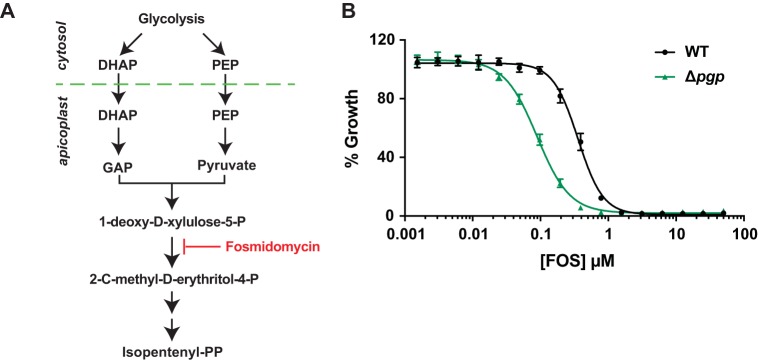
Loss of PGP leads to increased sensitivity to fosmidomycin. (A) DHAP and phosphoenolpyruvate, derived from glycolysis, are imported into the apicoplast for synthesis of isoprenoids via the 1-deoxy-d-xylulose 5-phosphate/2-*C*-methyl-d-erythritol 4-phosphate pathway. Fosmidomycin is a competitive inhibitor of the first committed enzyme in this pathway, and its efficacy is decreased when glycolytic flux is increased (26). (B) Synchronized WT- and Δ*pgp*-infected RBCs were treated with increasing concentrations of fosmidomycin for 72 h, and the growth percentage was determined by flow cytometry following SYTO61 labeling. Data are presented as the means ± SEM from three independent experiments performed on different days.

**FIG 6:**
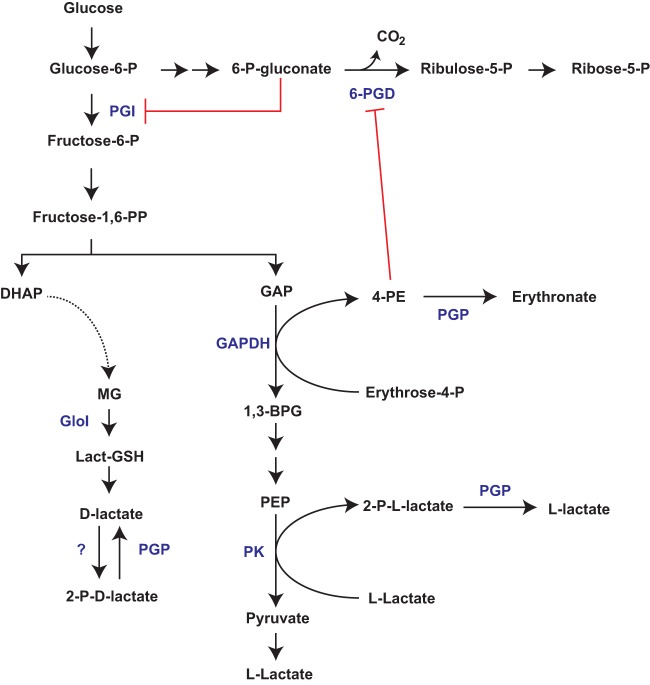
PGP-mediated metabolite repair maintains flux through glycolysis and the pentose phosphate pathway. PGP dephosphorylates 2-phospholactate (produced by an unspecified kinase[s] or a putative by-product of pyruvate kinase) and 4-phosphoerythronate (4-PE; a putative by-product of GAPDH). Loss of PGP leads to accumulation of 4-PE and partial inhibition of 6-PGD, with concomitant accumulation of 6-phosphogluconate and inhibition of enzymes in upper glycolysis. The PGP metabolic phenotype can be recapitulated by partial knockdown of 6-PGD. Abbreviations: glucose-6-phosphate isomerase (PGI), pyruvate kinase (PK), glyceraldehyde-3-phosphate dehydrogenase (GAPDH), 6-phosphogluconate dehydrogenase (6-PGD), glyoxylase I (GloI), phosphoglycolate phosphatase (PGP).

Δ*pgp* parasites appear to undergo reduced glycolytic flux due to a domino effect, wherein 4-PE levels accumulate and inhibit 6-PGD, leading to a build-up of the 6-PGD substrate, 6-phosphogluconate, which then inhibits the glycolytic enzyme glucose-6-phosphate isomerase (PGI). This metabolic blockade results in reduced levels of DHAP (Fig. 1C). DHAP and other triose-phosphates are catabolized in the glycolytic pathway and imported into the apicoplast, where they are used for synthesis of 1-deoxy-d-xylulose-5-P, the first committed intermediate in isoprenoid biosynthesis. Conversion of 1-deoxy-d-xylulose-5-P into 2-*C*-methyl-d-erythritol-4-P is inhibited by fosmidomycin, a potent antimalarial (Fig. 5A) (22). We hypothesized impaired glycolytic flux in the Δ*pgp* mutant could lead to reduced isoprenoid biosynthesis and increased sensitivity to fosmidomycin. Consistent with this hypothesis, we found that the sensitivity of Δ*pgp* parasites to fosmidomycin increased 4-fold compared to that of WT parasites (50% effective concentration [EC_50_]: WT, 358 ± 14 nM; Δ*pgp* line, 89 ± 9.8 nM; *P = *0.001) (Fig. 5B). These findings indicate that elevated 4-PE in Δ*pgp* parasites reduces glycolytic flux (Fig. 6), which limits isoprenoid biosynthesis and increases the potency of fosmidomycin.

## DISCUSSION

All eukaryotic and prokaryotic cells express members of the HAD family of metabolite phosphatases, although the functions of these proteins *in vivo* are poorly defined. There is increasing evidence that HAD enzymes have important roles in maintaining intracellular levels of phosphorylated intermediates and regulating metabolic fluxes. In this study, we provide evidence that the P. falciparum HAD family member PGP is a metabolic repair enzyme with at least two functions: dephosphorylation of the potential dead-end metabolite 2-phospholactate and removal of 4-PE (via dephosphorylation), a side product of glycolysis and potent inhibitor of enzymes in central carbon metabolism.

Targeted disruption of PGP resulted in the accumulation of both 2-phospholactate and 4-PE. 4-PE can be generated by the glycolytic enzyme GAPDH, acting upon the nonstandard substrate erythrose-4-P (an intermediate of the pentose phosphate pathway) (11) (Fig. 6). 4-PE is a potent inhibitor of 6-PGD *in vitro* (11, 23), and it was proposed that this can lead to blockage of the oxidative arm of the PPP (11). In P. falciparum, loss of PGP resulted in a 9-fold (±1.6) accumulation of 4-PE, and 4-PE was capable of >75% inhibition of 6-PGD at 100 μM. However, ^13^C-glucose labeling experiments indicated increased flux through the oxidative PPP in the Δ*pgp* line. While other interpretations are possible, these findings suggested that 4-PE inhibition of 6-PGD leads to compensatory metabolic responses that result in increased oxidative PPP flux. In support of this conclusion, we show that depletion of 6-PGD recapitulated the metabolic phenotype observed in PGP-disrupted parasites, leading to increased flux through the oxidative PPP (as indicated by elevated NADPH levels) and the downstream product ribose/ribulose-5-phosphate. We hypothesize that accumulation of 6-phosphogluconate leads to inhibition of the glycolytic enzyme PGI and redirection of the carbon flux into the oxidative PPP. Interestingly, partial inhibition of 6-PGD in Escherichia coli has also recently been shown to lead to a compensatory increased flux into the oxidative PPP through inhibition of upper glycolysis (24). These findings highlight how the parasite coordinates metabolic regulation via metabolite repair. Specifically, PGP dephosphorylates 4-PE and loss of function leads to a domino effect, wherein 4-PE accumulation inhibits 6-PGD, which in turn inhibits PGI via 6-phosphogluconate accumulation and reduces glycolytic flux.

PGP also dephosphorylates 2-phospholactate. However, the origins of 2-phospholactate in the malaria parasite are less clear, and the impact (if any) of the accumulation of this metabolite on cellular metabolism remains unclear. l-Lactate is the major end product of glycolysis, and 2-phospho-l-lactate is a minor side product of the glycolytic enzyme pyruvate kinase, which normally converts phosphoenolpyruvate to pyruvate (11). In contrast, in P. falciparum and other eukaryotes that lack a d-lactate-dehydrogenase, d-lactate is produced exclusively via the methylglyoxal pathway. We propose that the 2-phospholactate pool is derived from both d-lactate and l-lactate sources. Incubation of WT and Δ*gloI* parasites with methylglyoxal led to a concomitant increase in 2-phospholactate levels. Similarly, incubation of P. falciparum-infected RBCs with d-lactate, but not l-lactate, elevates intracellular levels of 2-phospholactate. The latter result indicates that 2-phospho-d-lactate is synthesized directly from d-lactate via the action of an unidentified kinase. Interestingly, a recent study suggested that the P. berghei PGP selectively utilized 2-phospho-l-lactate over 2-phospho-d-lactate *in vitro* (15), raising the possibility that this enzyme acts on both enantiomers *in vivo* depending on their intracellular concentrations.

The role of 2-phospholactate in P. falciparum remains unclear. 2-Phospho-l-lactate has been shown to inhibit the bifunctional glycolytic enzymes PFKFB1 to -4, which have both phosphofructokinase (PFK) and fructose-2,6-biphosphatase activities and regulate the intracellular levels of the PFK allosteric regulator fructose-2,6-bisphosphate (11). Strikingly, P. falciparum lacks a PFKFB homologue, and P. falciparum PFK is insensitive to conserved metabolic inhibitors/activators (25), suggesting that 2-phospholactate has a different role in P. falciparum. Our results raise the possibility that 2-phospholactate is an intermediate in a futile cycle of d-lactate phosphorylation/dephosphorylation that could contribute to regulation of ATP levels and/or potentially play a role in allosteric regulation of other enzymes.

This work highlights the important role of HAD enzymes in regulating P. falciparum central carbon metabolism. The roles of two other P. falciparum HAD enzymes have also recently been examined (9, 10). HAD1, the first member of this class to be functionally characterized in P. falciparum, promiscuously dephosphorylates a range of glycolytic intermediates *in vitro*, including triose-, pentose-, and hexose-phosphates. Loss-of-function mutations in this protein lead to increased parasite resistance to fosmidomycin, and it was proposed that HAD1 has a role in negatively regulating glycolytic flux by removing glycolytic intermediates (9). Similarly, HAD2 dephosphorylates a range of glycolytic intermediates *in vitro* and is linked to negative regulation of glycolysis, with mutations of this enzyme leading to increased resistance to fosmidomycin (10). In contrast, PGP is a positive regulator of glycolysis by preventing the accumulation of 4-PE and subsequent inhibition of PGI by 6-phosphogluconate. Consistent with this role, we demonstrate that PGP disruption sensitizes parasites to fosmidomycin by reducing the production of glycolytic intermediates for isoprenoid biosynthesis and increasing the competitive inhibition of fosmidomycin (26, 27). These findings highlight how metabolic repair underlies the parasite’s sensitivity and resistance to some antimalarials.

Our study illustrates the unanticipated complexity in parasite cellular metabolism that arises as a result of promiscuous activity of well-studied and conserved metabolic enzymes. These nonstandard reactions produce noncanonical metabolites that are not further catabolized by conventional pathways (dead-end metabolite) or are toxic by-products that need to be repaired for normal metabolic activity. In particular, control of 4-PE pool size via PGP appears to have an important role in regulating flux through glycolysis and the pentose phosphate pathway. Together, these findings highlight the role of HAD family members as important metabolic regulators of malaria parasite metabolism.

## MATERIALS AND METHODS

### CRISPR and pTEOE plasmid constructs.

GloI and PGP knockout constructs were cloned using the CRISPR/Cas9 system (18). Briefly, the guide RNA (gRNA) and two homology arms flanking the human dihydrofolate reductase (DHFR) cassette were cloned sequentially into the pL7-CS plasmid. The gRNAs were synthesized and cloned into pL7 using BtgZI restriction sites. Each homology arm sequence was amplified by PCR (CloneAmp; Clontech) from freshly prepared NF54 genomic DNA (isolate II genomic DNA kit; Bioline) and inserted into pL7 at specific restriction sites (homology arm 1, SpeI/AflII; homology arm 2, EcoRI/NcoI) using InFusion cloning (Clontech). pUF1-Cas9 was used unmodified (18). Plasmid stocks were prepared from Maxipreps (Macherey-Nagel). All sequences of interest were confirmed by Sanger sequencing (AGRF). Oligonucleotides used to generate and validate these constructs are presented in Table S2 in the supplemental material.

10.1128/mBio.02060-19.9 TABLE S2 Oligonucleotide list for generating PGP- and GloI-disrupted parasite lines. Oligonucleotides are presented by section. For the CRISPR and pTEOE cloning sections, InFusion oligonucleotides were designed (*). Letters in uppercase are nucleotides that are part of the plasmid backbone, and letters in lowercase are nucleotides that are part of the gene of interest. For GloI_HA1_rev_AflII, the nucleotides in blue are mutated nucleotides. Download Table S2, PDF file, 0.1 MB. Copyright © 2019 Dumont et al. 2019 Dumont et al. This content is distributed under the terms of the Creative Commons Attribution 4.0 International license.

For the localization of PGP, the coding sequence of PGP was amplified by Phusion PCR (NEB) from freshly prepared NF54 genomic DNA and inserted into the pTEOE-GFP plasmid (28) using XhoI and AvrII restriction sites and InFusion cloning (Clontech). To stably integrate the overexpression construct randomly into the genome, the pHTH helper plasmid expressing the *piggyBac* transposase system was used (29). Plasmid stocks were prepared from Midipreps (Macherey-Nagel). All sequences of interest were confirmed by Sanger sequencing (AGRF). Oligonucleotides used to generate and validate these constructs are presented in Table S2.

Expression of 6-PGD-HA *glmS* containing two LoxP sites for inducible disruption of the product 6-PGD was achieved using CRISPR/Cas9. The guide oligonucleotides SC101 and SC102 were designed to induce a double-stranded break at position 782 within PF3D7_1454700 and were InFusion cloned into pUF-Cas9G as described previously (18, 30). A 6-PGD replacement template was synthesized by GeneArt, containing ∼700 bases of homology upstream of the PAM site, an artificial intron containing a LoxP site, a recodonized version of the remaining coding sequence of PF3D7_1454700, 3× HA tag, 3× stop codon, and a second LoxP site (Table S3). This construct was flanked by BglII and SpeI restriction sites and was ligated into the pSBP-HA-glmS plasmid (replacing the SBP insert and referred to subsequently as p6-PGD-HA-glmS; T4 ligase; NEB). The 3′ homology arm was generated by PCR using oligonucleotides SC103 and SC104 and InFusion cloned into p6-PGD-HA-glmS using the restriction sites EcoRI and KasI. Correct clones were identified as described above via Sanger sequencing using oligonucleotides SC105 (3′ homology arm), SC106 (rescue template), and SC107 (guide insertion into pUF-Cas9G). Oligonucleotides used to generate and validate these constructs are presented in Table S3.

10.1128/mBio.02060-19.10 TABLE S3 Oligonucleotide list and rescue template used for generating the DiCre-6-PGD parasite line Download Table S3, PDF file, 0.02 MB. Copyright © 2019 Dumont et al. 2019 Dumont et al. This content is distributed under the terms of the Creative Commons Attribution 4.0 International license.

### P. falciparum parasite culture and transfections.

P. falciparum 3D7 and NF54 parasites were cultured in O^+^ human red blood cells (Australian Red Cross) in RPMI-HEPES–GlutaMAX (Gibco Life Technologies) with 0.25% AlbuMAX (Gibco Life Technologies), 5% human serum (Australian Red Cross), 10 mM glucose, 50 μM hypoxanthine, and gentamicin (Sigma) at 1 to 4% hematocrit at 37°C in 5% CO_2_, 1% O_2_, nitrogen balance (Coregas). Cultures were monitored by Giemsa smears. Sorbitol (31) or magnetic enrichments (32) were used to maintain synchronous cultures. Cultures were regularly assessed for mycoplasma contamination (Mycoalert; Lonza), and all experiments reported here were on mycoplasma-free lines.

For knockout constructs, NF54 and 3D7 parasites were transfected at ring stage using 75 μg of each plasmid (pL7 and pUF-1-Cas9). For overexpression constructs, NF54 parasites were transfected at ring stage using 100 μg of pTEOE and 50 μg of pHTH as previously described (33). DNA-cytomix was electroporated into red blood cells at 310 V and 950 μF as previously described (34). Parasites were maintained on 5 nM WR99210 (Sigma-Aldrich) selection at all times. Knockout parasites were also selected on 1.5 μM DSM-1 (MR4) for the first 5 days posttransfection. On the first day, parasites were observed by Giemsa smears (16 to 25 days posttransfection), the cultures were separated in half, and 5-fluorocytosine (negative selection) was added to recovered transfectants until healthy rings returned, 2 to 6 days later (35). Each of the generated parasite lines was used as a mixed population (as transfection efficiency with the CRISPR/Cas9 system was reported to be very high [36]). Validation of successful integration was confirmed by PCR on knockout-NF54 genomic DNA (Lucigen), using oligonucleotide pairs that were specific to the integrated cassette and the genomic DNA. Oligonucleotides used for this purpose are presented in Table S2.

For generation of the 6-PGD inducible disruption parasite line, 100 μg of p6-PGD-HA-glmS plasmid was linearized using the BglI, BglII, and PvuI restriction sites and was electroporated into ring-stage 3D7-DiCre parasites in conjunction with the pUF-Cas9G plasmid (100 μg). Parasites were maintained on Blasticidin S and WR99210 and integration confirmed using oligonucleotides SC108 and SC109 (for nonintegration) and SC108 and SC110 (for integration; Table S3).

### P. falciparum parasite growth assay.

The parasitemia of synchronized ring cultures (0.8% hematocrit, 0.8% parasitemia) was assessed by flow cytometry each day for 13 days, and identical dilutions between WT- and knockout-infected RBCs were applied regularly. Nucleic acids were stained with SYTO 61 (Invitrogen), and parasitemia was measured on a FACS Canto II (BD Biosciences). Data were analyzed using FlowJo software (BD Biosciences). Raw counts were normalized to the day 13 WT values, and the dilutions made throughout the experiment were corrected for. For DiCre-6-PGD parasites and the DiCre parental line, growth was assessed in the presence or absence of 100 nM rapamycin (Santa Cruz) and 2.5 mM glucosamine, and parasitemia was determined every 48 h using flow cytometry as described above.

### d-Lactate *in vitro* assay.

The concentration of d-lactate excreted from uninfected and NF54-infected RBCs was measured using a d-lactate assay kit (Cayman Chemical). All conditions were incubated for 26 to 29 h (ring to trophozoite stage) in complete RPMI medium. Medium was collected, deproteinized, and measured by following the manufacturer’s instructions. After incubation for 30 min at 37°C, the plate was read at 590 nm (excitation wavelength, 544 nm) on a FluoStar Omega plate reader (BMG Labtech) and analyzed with the Omega data software.

### *In vitro* enzymatic activity assays.

Infected RBCs were magnetically enriched at the trophozoite stage (>95% parasitemia; MAGNEX cell separator; Colebrook Bioscience) and lysed in 0.1% saponin buffer. After three PBS washes and counting of isolated parasites, cell pellets were snap-frozen in liquid nitrogen and stored at −80°C. Pellets were resuspended in 200 μl per 1 × 10^8^ cells of pH 7.4 lysis buffer (5 mM HEPES, 2 mM DTT, protease inhibitor). Enzymatic reaction mixtures were set up in a 50:50 ratio of cell lysate to reaction buffer. For measuring glyoxalase activity, the pH 6.8 reaction buffer was composed of 100 mM Tris HCl, 5 mM NH_4_Cl, 2 mM MgCl_2_, 2 mM ATP, 2 mM methylglyoxal (MG), and 2 mM reduced glutathione. The control reaction consisted of the reaction buffer without MG (−MG condition) and was used as the baseline for data normalization. Enzymatic reactions were set up at 37°C, and samples were collected in technical duplicates at 0, 5, 10, 30, and 60 min.

6-PGD activity was measured as described above using a pH 6.8 reaction buffer composed of 100 mM Tris HCl, 5 mM NH_4_Cl, 2 mM MgCl_2_, 1 mM 6-phosphogluconate, 1 mM NADP, and 1 mM 4-PE (as annotated). Enzymatic reactions were set up at 37°C, incubated for 60 min, and collected in technical duplicates at 0 and 60 min. Samples were extracted for GC-MS analysis (described below).

### GC-MS sample extraction and derivatization.

*In vitro* assay samples and cell pellets were extracted on ice with 100 μl chloroform and 400 μl 3:1 methanol-H_2_O containing 1 nM scyllo-inositol (internal standard). Extracts were made biphasic by addition of water (200 μl) and samples mixed thoroughly and centrifuged (14,000 × *g*, 5 min, room temperature). The upper aqueous phase was transferred into a new tube and dried in a SpeedVac (Savant). Dried polar metabolite extracts were washed in 600 μl 90% methanol, and 30-μl volumes were transferred into glass vial inserts (Agilent) (1/20 dilution) and dried (SpeedVac). Samples were further dried by addition of methanol (50 μl) and then derivatized with 20 μl of 20 mg/ml methoxyamine (Sigma-Aldrich) made up in pyridine (Sigma-Aldrich) at room temperature for ∼12 h. Samples were converted to their trimethylsilyl derivatives by addition of N,O-bis(trimethylsilyl) trifluoroacetamide (BSTFA) plus 1% trimethylchlorosilane (TMCS) solution (20 μl; Supelco) and then analyzed by GC-MS using a BD5 capillary column (30 m by 250 μm by 0.25 μm; J&W Scientific) on a Hewlett Packard 6890 system (5973 EI-quadrupole MS detector) as previously described (37). Briefly, the oven temperature gradient was 70°C (1 min); 70°C to 295°C at 12.5°C/min, 295°C to 320°C at 25°C/min; and 320°C for 2 min. MS data were acquired using scan mode with an *m/z* range of 50 to 550, threshold of 150, and scan rate of 2.91 scans/s. GC retention time and mass spectra were compared with authentic standards analyzed in the same batch for metabolite identification.

For estimating the intracellular concentration of 4-PE, a known concentration of 4-PE was measured via GC-MS alongside a metabolite extract from 1 × 10^8^ purified infected trophozoite-stage RBCs. The 4-PE peaks detected in both the pure standard and biological sample were integrated, and the final concentration of 4-PE in the biological sample was determined. The intracellular concentration of 4-PE was determined using the known number of cells within the extract (1 × 10^8^) and the estimated intracellular volume of an infected RBC (75 fl) (38).

### ^13^C-glucose labeling and LC-MS analysis.

Synchronized trophozoite cultures were magnetically enriched using a MAGNEX cell separator (Colebrook Bioscience). Purified cells (5 × 10^7^ cells/sample) were incubated for 30 min at 37°C in RPMI containing unlabeled glucose, RPMI containing 11 mM ^13^C_1,2_-glucose (Cambridge Isotopes) (termed fully labeled), or 11 mM ^13^C-U-glucose mixed 1:1 with complete RPMI containing 11 mM unlabeled glucose (Sigma). Aliquots of cell cultures (1 ml) were centrifuged (14,000 × *g*, 30 s) and washed in 1 ml ice-cold PBS, and pellets were extracted for GC-MS analysis for ^13^C_1,2_-glucose labeled samples (as described above) or extracted for LC-MS analysis (^13^C-U-glucose samples). For DiCre-6-PGD analysis, infected cultures were maintained under standard culturing conditions in the presence or absence of 100 nM rapamycin (Santa Cruz) and 2.5 mM glucosamine (Sigma) for 24 h prior to magnetic enrichment at trophozoite stage.

LC-MS samples were resuspended in 100 μl of 80% acetonitrile (Burdick & Jackson) containing the internal standard 1 μM 4-^13^C,^15^N-aspartate. After centrifugation (14,000 × *g*, 5 min, 4°C), supernatants were transferred into glass vials and LC-MS analysis performed as described previously (37), with the following modifications. Metabolite samples were separated on a SeQuant ZIC-pHILIC column (5 μm, 150 by 4.6 mm; Millipore) using a binary gradient with a 1200 series HPLC system (Agilent), with solvent A being water with 20 mM ammonium carbonate and solvent B 100% acetonitrile. The gradient ran linearly (at 0.3 ml/min) from 80 to 20% solvent B from 0.5 to 15 min and then 20 to 5% between 15 and 20 min before returning to 80% at 20.5 min and kept at 80% solvent B until 29.5 min. MS detection was performed on an Agilent Q-TOF mass spectrometer 6545 operating in negative electrospray ionization (ESI) mode. The scan range was 80 to 1,200 *m/z* between 2 and 25 min at 0.9 spectra/second. An internal reference ion solution was continually run (isocratic pump at 0.2 ml/min) throughout the chromatographic separation to maintain mass accuracy. Other LC parameters were autosampler temperature of 4°C and injection volume of 10 μl. Data were collected in centroid mode with Mass Hunter Workstation software (Agilent).

### Mass spectrometry data analysis.

GC-MS data were processed using ChemStation (Agilent) or the in-house software package DExSI (39), and metabolites of interest were compared to authentic standards. LC-MS.d files were converted to .mzXML files using MS Convert and analyzed using MAVEN (40). Following alignment, peaks were extracted with a mass tolerance of <10 ppm. Untargeted comparative profiling was performed to generate a list of *m/z* features of interest. The *m/z* value of each peak of interest was then queried against the METLIN metabolite database for only M-H adducts with a 10-ppm mass tolerance. Peaks of interest were positively identified using their exact mass and retention time (compared to a standards library of 150 compounds run the same day).

### Synthesis of sodium phospholactate.

Pearlman’s catalyst [20% Pd(OH)_2_-C, 135 mg] was added to a solution of phospho(enol)pyruvic acid monosodium salt monohydrate (40 mg, 192 μmol; Sigma) in tetryhydrofuran-methanol (MeOH)-acetic acid 1:1:1 (3 ml), and the mixture was stirred at room temperature under an atmosphere of H_2_ (4 × 10^5^ mPa) for 48 h. The reaction mixture was diluted with MeOH (20 ml), filtered through Celite, and evaporated at 10^5^ mPa on a rotary evaporator at 40°C. The residue was dissolved in deionized water, and the solution was passed through a C_18_ Sep-Pak. The eluent was evaporated at 10^5^ mPa on a rotary evaporator at 40°C, and the residue was dried at 100 mPa at room temperature for 24 h, affording d/l-phospholactate monosodium salt hydrate (40 mg) as a viscous oil. High-resolution MS (ESI-time of flight) calculated for C_3_H_4_O_6_P^−^ [M – Na]^−^, 168.9907 *m/z*; found, 168.9917 *m/z*.

### Synthesis of cyclohexylammonium 4-phospho-d-erythronate.

Br_2_-saturated H_2_O (616 μl, ∼35 mg/ml, 135 μmol) was added to a stirred mixture of d-erythrose-4-phosphate (12.0 mg, 54.0 μmol; Sigma) in aqueous Na_2_CO_3_ (400 μl, 0.405 M, 162 μmol) at room temperature. After 1 h, excess bromine was removed by sparging for 2 h with N_2_. The resultant mixture was passed down a column containing Amberlite Ag50 (acid form, 2-ml bed volume, ∼1.7 mM H^+^ per ml), which was rinsed with H_2_O (1.5 ml three times). Cyclohexylamine (39 μl, 0.324 mmol) was added to the eluted product, and the volatiles were removed by rotary evaporation and further drying under an N_2_ stream overnight. The crude material was purified on a Shimadzu 2020 LC-MS instrument, using an ACE Excel 5 Super C_18_ column (150 mm by 2.1 mm) with 0.1% formic acid (solvent A) and methanol (solvent B). A linear gradient was performed from 80% to 10% across 15 min, and elution of 4-PE was determined using the theoretical exact mass of 4-PE. Separation of 4-PE from contaminants was monitored using the MS in full scan mode and with UV detection. Verification of the purified 4-PE was performed by rerunning the collected aliquot on the instrument and confirming no other masses or UV peaks were observed (above background). Expected [M – H]^−^ was 214.9962 *m/z*; observed was 214.9968 *m/z*.

### Fluorescence microscopy.

Glass coverslips were incubated with 0.1 mg/ml lectin from *Phaseolus vulgar* (PHAE) (Sigma-Aldrich) for 30 min at 37°C in a humidity chamber. After 3 washes in PBS, infected RBCs (3% hematocrit) were incubated for 10 min on the coverslip. Nuclei were stained using 2 μg/ml 4′,6-diamidino-2-phenylindole (DAPI) for 10 min. Slides were mounted in *p*-phenylenediamine antifade. Images were taken on a Delta Vision elite restorative widefield deconvolution imaging system (GE Healthcare) using a 100× UPLS Apo objective (1.4 numeric aperture; Olympus) lens under oil immersion. The following emission/excitation filter sets were used: DAPI, excitation at 390/18 and emission at 435/48; fluorescein isothiocyanate, excitation at 475/28 and emission at 523/26. Images were deconvoluted using Softworx 5.0 (GE Healthcare) and analyzed using ImageJ (NIH).

### Protein analysis by Western blotting.

Trophozoite parasites were isolated by addition of 0.05% saponin in the culture media and spun down at 3,750 × *g* for 5 min. Pellets were washed twice in ice-cold PBS containing complete protease inhibitors (Roche) and resuspended in PBS or RIPA buffer containing protease inhibitors. RIPA buffer samples were incubated on ice for 10 min and centrifuged at 16,000 × *g* for 10 min. The supernatant was collected and placed in a fresh tube. The saponin pellet (PBS) and the RIPA (supernatant) samples were mixed with Bolt 4× LDS and 10× reducing agent (Invitrogen) and heated at 85°C for 10 min prior to SDS-PAGE on 4 to 12% BisTris gels separated in 1× morpholinepropanesulfonic acid running buffer (Invitrogen). Proteins were transferred onto nitrocellulose membranes using the iBlot 2 transfer system (Invitrogen), and membranes were blocked in 3.5% skim milk for at least 1 h at room temperature. Membranes were probed with mouse anti-GFP (1:1,000; Roche) and rabbit anti-GAPDH (1:1,000) (41) primary antibodies. Secondary antibodies were horseradish-peroxidase (HRP) conjugated goat anti-mouse and anti-rabbit (1:20,000; Promega). Membranes were incubated with Clarity ECL substrate (Bio-Rad) and imaged on a ChemiDoc MP system (Bio-Rad). For DiCre-6-PGD Western blotting, rat anti-HA (1:1,000; Roche) and goat HRP anti-rat (1:10,000) were used to probe membranes generated as described above. Mouse anti-BiP (1:5,000) was used to confirm equal loading between conditions.
