# Supplementary material for: The Metabolite Repair Enzyme Phosphoglycolate Phosphatase Regulates Central Carbon Metabolism and Fosmidomycin Sensitivity in Plasmodium falciparum
Source: mBio. 2019 Dec 10;10(6):e02060-19. doi: 10.1128/mBio.02060-19 (PMC6904873; doi:10.1128/mBio.02060-19)
Supplement: FIG S1 [file mBio.02060-19-sf001.pdf]

Figure S1

```
                                                           I
P. falciparum MALIYSSDKKDDDIINVEKKYESFLKEWNLNKMINSKDLCLEFDVFFFDCDGVLWHGNEL 60
Yeast         MT----AQ---QGV------------PIKITNKEIAQEFLDKYDTFLFDCDGVLWLGSQA 41
Mouse         MAE---AEAGGDEA------------RCVRLSAERAKLLLAEVDTLLFDCDGVLWRGETA 45
Human         MAA---AEAGGDDA------------RCVRLSAERAQALLADVDTLLFDCDGVLWRGETA 45
              *:    ::   :                   .   :: :  . *.::******** *.

                                 II
P. falciparum IEGSIEVINYLLREGKKVYFITNNSTKSRASFLEKFHKLGFTNV----KREHIICTAYAV 116
Yeast         LPYTLEILNLLKQLGKQLIFVTNNSTKSRLAYTKKFASFGIDVK-----EEQIFTSGYAS 96
Mouse         VPGAPETLRALRARGKRLGFITNNSSKTRTAYAEKLRRLGFGGPVGPEAGLEVFGTAYCS 105
Human         VPGAPEALRALRARGKRLGFITNNSSKTRAAYAEKLRRLGFGGPAGPGASLEVFGTAYCT 105
              :  : * :. *   **:: *:****:*:* :: :*:  :*:          .:: :.*.

P. falciparum TKYLYDKEEYRLRKKKIYVIGEKGICDELDASNLDWLGGSNDNDKK---IILKDDLEIIV 173
Yeast         AVYIRDFLKLQPGKDKVWVFGESGIGEELKLMGYESLGGADSRLDTPFDAAKSPFLVNGL 156
Mouse         ALYLRQRLAGVPD-PKAYVLGSPALAAELEAVGVTSVGVGPDVL---HGDGPSDWLAVPL 161
Human         ALYLRQRLAGAPA-PKAYVLGSPALAAELEAVGVASVGVGPEPL---QGEGPGDWLHAPL 161
              : *: :         * :*:*. .:  **.  .   :* . .             *   :

P. falciparum DKNIGAVVVGIDFNINYYKIQYAQLCINELNAEFIATNKDATGNFTSKQKWAGTGAIVSS 233
Yeast         DKDVSCVIAGLDTKVNYHRLAVTLQYLQKDSVHFVGTNVDSTFPQKG-YTFPGAGSMIES 215
Mouse         EPDVRAVVVGFDPHFSYMKLTKAVRYLQQPDCLLVGTNMDNRLPLENGRFIAGTGCLVRA 221
Human         EPDVRAVVVGFDPHFSYMKLTKALRYLQQPGCLLVGTNMDNRLPLENGRFIAGTGCLVRA 221
              : :: .*:.*:* :..* ::  :   ::: .  ::.** *      .     *:*.:: :

                          III                        IV
P. falciparum IEAVSLKKPIVVGKPNVYMIENVLKDLNIHHSKVVMIGDRLETDIHFAKNCNIKSIL-VS 292
Yeast         LAFSSNRRPSYCGKPNQNMLNSIISAFNLDRSKCCMVGDRLNTDMKFGVEGGLGGTLLVL 275
Mouse         VEMAAQRQADIIGKPSRFIFDCVSQEYGINPERTVMVGDRLDTDILLGSTCSLKTIL-TL 280
Human         VEMAAQRQADIIGKPSRFIFDCVSQEYGINPERTVMVGDRLDTDILLGATCGLKTIL-TL 280
              :   : ::    ***.  ::: : .  .:. .:  *:****:**: :.   .:   * .

P. falciparum TGVTNAN-IYLN-----HNSLNIHPDYFMKSISELL-------   322
Yeast         SGIETEE------RALKISHDYRPRKFYIDKLGDIYTLTNNEL   312
Mouse         TGVSSLEDVKSNQESDCMFKKKMVPDFYVDSIADLLPALQG--   321
Human         TGVSTLGDVKNNQESDCVSKKKMVPDFYVDSIADLLPALQG--   321
              :*: .                   *.:::..:.::
```
