# Supplementary figures and images for: The Metabolite Repair Enzyme Phosphoglycolate Phosphatase Regulates Central Carbon Metabolism and Fosmidomycin Sensitivity in Plasmodium falciparum
Source: mBio. 2019 Dec 10;10(6):e02060-19. doi: 10.1128/mBio.02060-19 (PMC6904873; doi:10.1128/mBio.02060-19)

Figure S2

a)

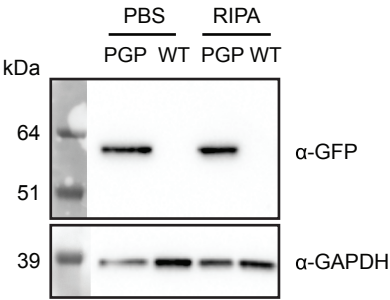

b)

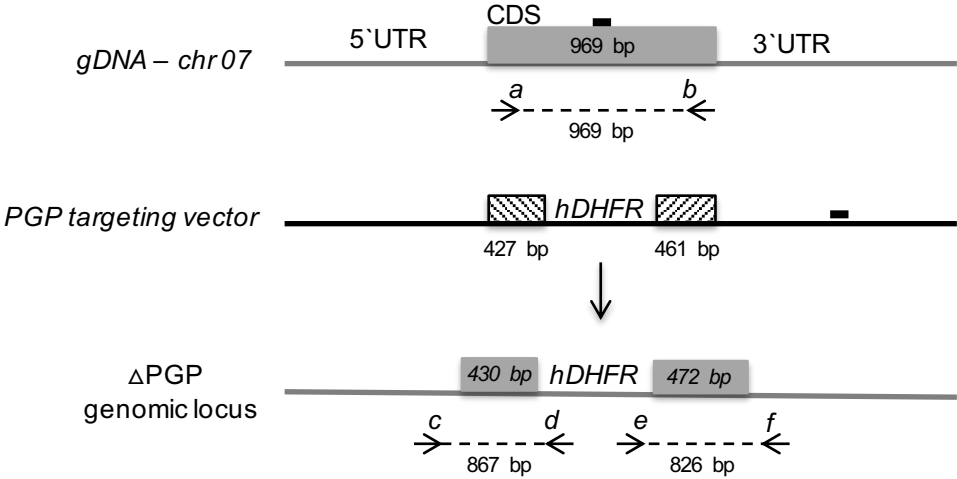

c)

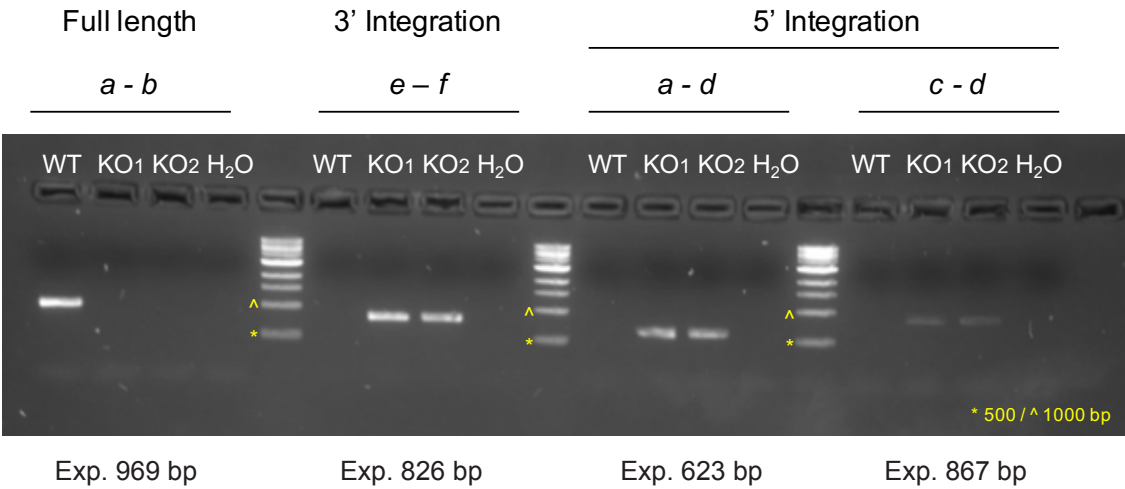

Supplement: FIG S2 [file mBio.02060-19-sf002.pdf]

Figure S3

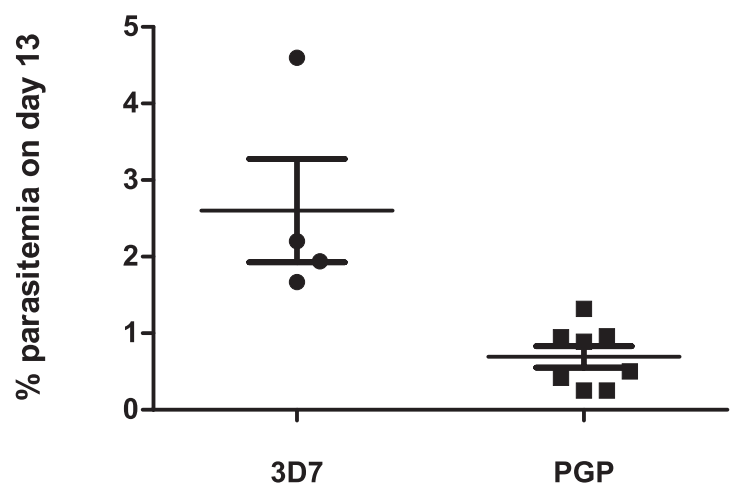

Supplement: FIG S3 [file mBio.02060-19-sf003.pdf]

Figure S4

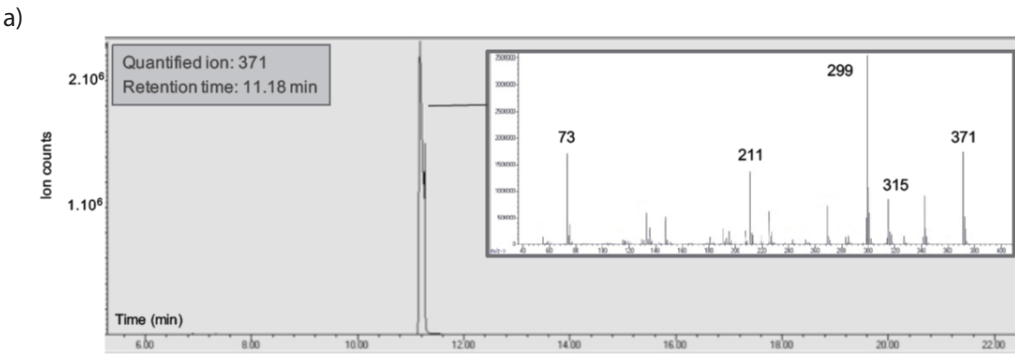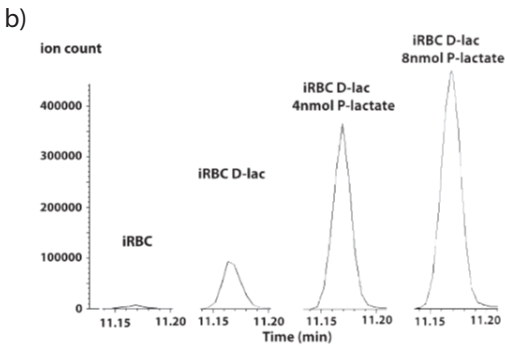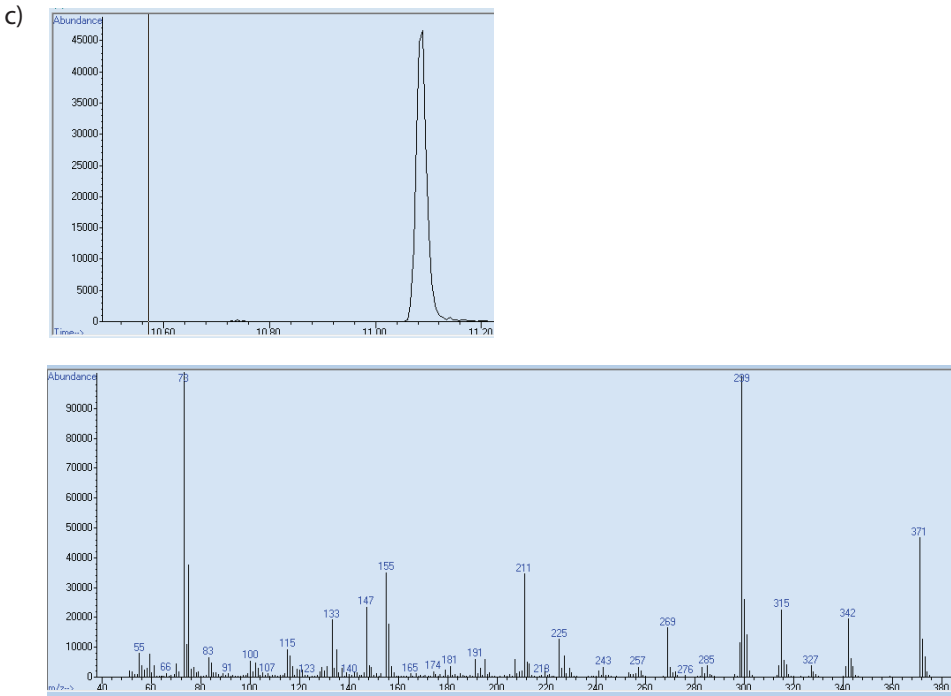

Supplement: FIG S4 [file mBio.02060-19-sf004.pdf]

Figure S5

a)

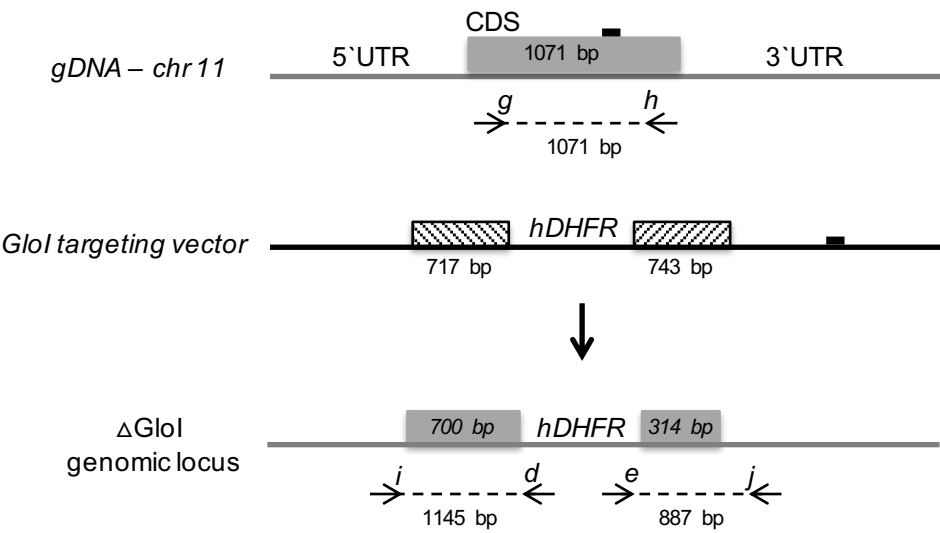

b)

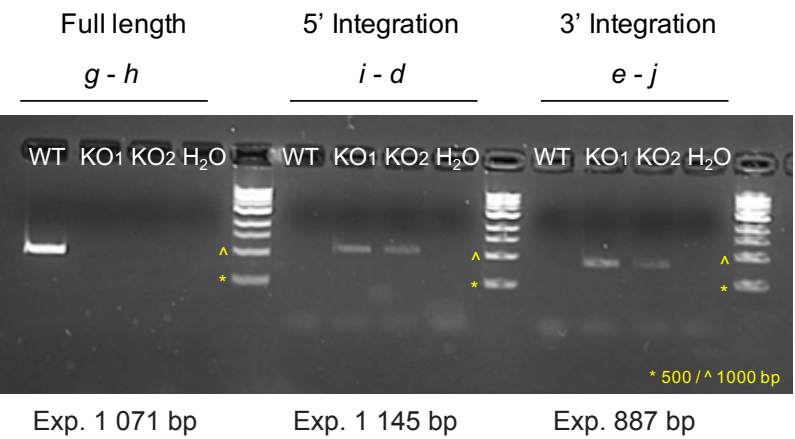

Supplement: FIG S5 [file mBio.02060-19-sf005.pdf]

Figure S6

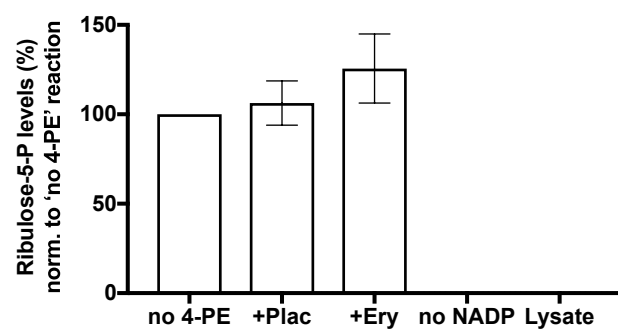

Supplement: FIG S6 [file mBio.02060-19-sf006.pdf]

Figure S7

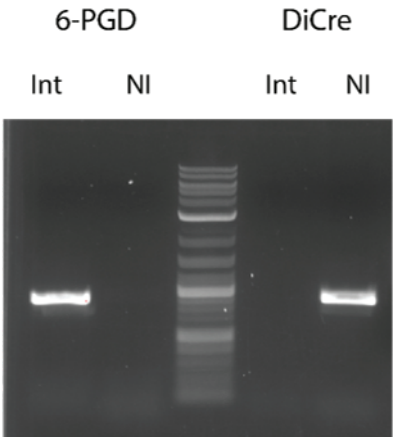

Supplement: FIG S7 [file mBio.02060-19-sf007.pdf]
