# Supplementary material for: The Metabolite Repair Enzyme Phosphoglycolate Phosphatase Regulates Central Carbon Metabolism and Fosmidomycin Sensitivity in Plasmodium falciparum
Source: mBio. 2019 Dec 10;10(6):e02060-19. doi: 10.1128/mBio.02060-19 (PMC6904873; doi:10.1128/mBio.02060-19)
Supplement: TABLE S1 [file mBio.02060-19-st001.pdf]

Table S1. Repertoire of the metabolites-of-interest and analytical GC-MS features

	Quantified ion	Retention time (min)
**D-lactate**	117	5.80
**Phospholactate**	371	11.52
**4-Phosphoerythronate**	357	15.09
**Ribulose-5-P**	357	16.11
**Ribose-5-P**	459	16.03
