# Supplementary material for: The Metabolite Repair Enzyme Phosphoglycolate Phosphatase Regulates Central Carbon Metabolism and Fosmidomycin Sensitivity in Plasmodium falciparum
Source: mBio. 2019 Dec 10;10(6):e02060-19. doi: 10.1128/mBio.02060-19 (PMC6904873; doi:10.1128/mBio.02060-19)
Supplement: TABLE S2 [file mBio.02060-19-st002.pdf]

Table S2. Primer list

**CRISPR Cloning***	
GloI_gRNA_fw	TAAGTATATAATATTtgcatgtgcagaaaataataGTTTTAGAGCTAGAA
GloI_gRNA_rev	TTCTAGCTCTAAAACtattattttctgcacatgcaAATATTATATACTTA
GloI_HA1_fw_SpeI	GGGGAGGACTAGTcatatatattttacaatatggcacaa
GloI_HA1_rev_AflII	TTACAAAATGCTTAAGctatcatttc**t**tt**g**tt**g**ttttctgca
GloI_HA2_ fw_EcoRI	TTAAATCTAGAATTCcatatcaaacagatgaagattatgaa
GloI_HA2_rev_NcoI	TTTACCGTTCCATGGagttaattataacaaatcgatataaaaaa
PGP_gRNA_fw	TAAGTATATAATATTgcatctaatcttgattggttGTTTTAGAGCTAGAA
PGP_gRNA_rev	TTCTAGCTCTAAAACaaccaatcaagattagatgcAATATTATATACTTA
PGP_HA1_fw_SpeI	GGGGAGGACTAGTgctttaatttattcgagtgataaaaaa
PGP_HA1_rev_AflII	TTACAAAATGCTTAAGcatcacatattcctttttctccaat
PGP_HA2_ fw_EcoRI	TTAAATCTAGAATTCagatgatttagaaattattgtagataa
PGP_HA2_rev_NcoI	TTTACCGTTCCATGGccgatatggatttcataaaataatca
**CRISPR Sequencing**	
gRNA_a	GCATAATTTTTCCTATATGCACATA
gRNA_b	GGTAGCCTTAAAAACTTCATTATATT
HA1_c	CATGCAAAAATTTACTATAATATTA
HA1_d	TACAAGTATATATTTTGTTTCTATAA
HA2_e	CAATATGAACATAAAGTACAACATT
HA2_f	CGGTTGTCCCTTTGATAATAT
**CRISPR Integration**	
Int_*a*	atggctttaatttattcgagtgata
Int_*b*	ttataataattccgatatggatttcat
Int_*c*	cataggtacatacatatatatgtttata
Int_*d*	caagtatatattttgtttctataaattga
Int_*e*	caatatgaacataaagtacaacatt
Int_*f*	gagagtacacaaatataataacaaa
Int_*g*	atggcacaagaaatatcaaatttag
Int_*h*	ttattttgcaataaatgaagtgtcc
Int_*i*	gcttcatcttgaattgaaaagatt
Int_*j*	ttgttaaatgtatgtatatgtgaatatatg
**pTEOE cloning***	
pTEOE-PGP_fw_XhoI	CGATTTTTTCTCGAGatggctttaatttattcgagtgataaa
pTEOE-PGP_rev_AvrII	ATGTGCTGCACCTGGCCTAGGtaataattccgatatggatttcata
**pTEOE-sequencing**	(primers from Dr Natalie Spillman)
pTEOE-seq_fw	TACTTTAAAAAGAAATTTTCC
pTEOE-seq_rev	CAGAAAATTTGTGCCCATTAACATCA
