# Supplementary material for: The Metabolite Repair Enzyme Phosphoglycolate Phosphatase Regulates Central Carbon Metabolism and Fosmidomycin Sensitivity in Plasmodium falciparum
Source: mBio. 2019 Dec 10;10(6):e02060-19. doi: 10.1128/mBio.02060-19 (PMC6904873; doi:10.1128/mBio.02060-19)
Supplement: TABLE S3 [file mBio.02060-19-st003.pdf]

Table S3 – Oligos and rescue template for 6-PGD inducible disruption

Oligo Name	Sequence
SC101	TAAGTATATAATATTATTGCTGGAGCAAAAGGTACGTTTTAGAG<br>CTAGAA
SC102	TTCTAGCTCTAAAACGTACCTTTTGCTCCAGCAATAATATTATAT<br>ACTTA
SC103	ATTAAATCTAGAATTCGAAATGGACTATGCTTGAAGCTACC
SC104	TACCGCATCAGGCGCCCCACAAGGTGTGATAATTTCCTTC
SC105	CTTTTACAATATGAACATAAAGTACAACATTAATATATATAGC
SC106	GTTGTGTGGAATTGTGAGCGG
SC107	ATAATGTAAAAATAAAGGGTAAATTATTATTAAAAAATGTATAT<br>GTTATG
SC108	Cgtactttcctttaaggc
SC109	ATATATGTATCTATTTATTCAAAAGTCT
SC110	CAGCACACATAGTTGGA
Rescue Template	AGATCTTGGTGTTTATAATAGGACATATGAAAGAACAGAAGAAACAATGAAAA<br>GAGCAAAAGAAGAGAATTTGGTTGTTTATGGTTATAAAACAGTAGAAGAATTA<br>ATAAATAATTTGAAAAAACCAAGGAAAGTTATTTTATTAATCAAAGCAGGTCCA<br>GCTGTAGATGAGAATATTAGTAATATATTAAAACATTTTGAAAAAGGAGATATA<br>ATAATTGATGGTGGGAATGAATGGTATATTAATTCAGAAAGAAGAATAAAATT<br>ATGTAAAGAAAAAGATGTAGAATATTTAGCTATGGGTGTGAGTGGAGGTGAAG<br>CAGGTGCAAGATATGGTTGTTCATTTATGCCTGGTGGTTCTAAATATGCATATG<br>ATTGTGTGAAAGAAATATTAGAAAAATGTTCAGCTCAAGTTGGAAATTCTCCTT<br>GTGTTACTTATATAGGTCCAGGTTCCTCAGGGAATTATGTAAAAATGGTACATA<br>ATGGAATAGAATATGGAGATATGCAATTAATATCAGAAAGTTATGTAATTATGA<br>AACATATATTAAAATATGATAATCAGAAATTATCAGAAGTTTTTAATAAATGGA<br>ATGAAGGTATATTAAATTCTTACTTAATTGAAATTACTGCAAATATTCTTGCAAA<br>AAAAGATGACTTAACAAATAATTATTTAGTTGATATGATATTAGATATTGCTGG<br>AGCTAAGGTAAGATATAAAAGATAAATATATGAATAAAATATAAGACTTTTGA<br>ATAAATAGATACATATATATAATAACTTCGTATAATGTATGCTATACGAAGTTA<br>TTATATATATATATATATATATATATATATATATATATATATGTATTTATATATATTT<br>TTTTTTTTTTAGGGAACTGGCAAGTGGACAATGTTGGAGGCAACTGAGAGGGGA<br>ATACCATGTCCTACAATGTGTGCAGCACTTGACGCTAGGAACATTTCCGTATTT<br>AAGGAGCTTAGGACAAAGGCCGAGAGTAACTTCAACAAGGACAACATATTGAT<br>AGATCCTAACGAGGACCTTAACGACTTTGAGAACGACCTTCTTAACGCCTTGTA<br>CTGCTGTAAGATAATAAGTTATACACAGGGATTGTTCTTGCTTAAGCAGGTAAG<br>TGAGGAGATGAATTGGAAGCTTAACCTTGGTGAGATAGCAAGGATTTGGAGGG<br>GAGGATGTATAATTAGGGCAGTATTTCTTGACAGGATTGCCAACGCCTATAAGA<br>ACAACGAGAAGCTTGAGCTTCTTTTCCTTGACAACGAGTTTAGTGACGACATAA<br>AGAACAAGCTTCCTAGTCTTAGGAAGATAGTATTGATGGCAACTAAGTATAGTA<br>TACCAATTCCTGCTTTTAGTGCATCACTTGCTTACTTCCAGATGGTTACGAGTCA<br>GAACCTTCCATTGAACCTTGTCCAGGCTCAGCGTGACTACTTCGGAAGTCATAC<br>TTATAGGAGGACTGACAGGGAGGGTAACTATCATACTCTTTGGGCTGCCGCATA<br>CCCGTACGACGTCCCGGACTACGCTGGCTATCCCTATGATGTGCCCGATTATGC<br>GTATCCGTACGATGTTCCAGATTATGCCTAATGATAGATAACTTCGTATAATGT<br>ATGCTATACGAAGTTATACTAGT

Black = 6PGD 5’homology region upstream of PAM site

Red = artificial intron w 1st loxP site

Purple = recodonized remaining 6PGD coding sequence

Yellow = 3xHA, 3x Stop codon, 2nd loxP site

Blue = BglII and SpeI restriction sites
